# Soil conditioners promote cadmium reduction and selenium enrichment in rice: formulation process, effects, and mechanisms

**DOI:** 10.3389/fpls.2026.1887305

**Published:** 2026-08-20

**Authors:** Jingshang Xiao, Hongqian Hou, Jianhua Ji, Zhenzhen Lv, Xianjin Lan, Yiren Liu, Yifan Ma, Long Qian, Ying Liu, Qinlei Rong, Lijun Zhou, Yun Liu, Sijia Zuo, Zhixin Luo

**Affiliations:** 1 Institute of Soil Fertilizer and Resource Environment, Jiangxi Academy of Agricultural Sciences/National Engineering and Technology Research Center for Red Soil Improvement/Key Laboratory of Acidified Soil Amelioration and Utilization, Ministry of Agriculture and Rural Affairs, Nanchang, China; 2 Institute of Water Resources and Environmental Design, CSCEC AECOM Consultants Co., Ltd, Xian, China; 3 National Biopesticide Engineering Technology Research Center, Hubei Biopesticide Engineering Research Center, Hubei Academy of Agricultural Sciences, Wuhan, China; 4 Yichun City Selenium Resource Development and Utilization Center, Yichun, China; 5 College of Land Resources and Environment, Jiangxi Agricultural University, Nanchang, China; 6 National Red Soil Improvement Engineering Technology Research Center, Jiangxi Institute of Red Soil, Nanchang, China; 7 Jiangxi Purifeng Ecological Technology Co., Ltd., Nanchang, China

**Keywords:** cadmium, rice, selenium, soil, soil conditioners

## Abstract

Excessive accumulation of cadmium (Cd) in rice from a selenium (Se)-enriched area is a global environmental issue. Due to its widespread presence and potential transfer through the food chain, it poses a significant threat to human health. Se, as an essential micronutrient for humans, reduces the Cd uptake of rice and mitigates the toxicity of Cd. The application of optimized soil conditioners has emerged as a promising agronomic strategy to mitigate Cd accumulation in rice grains while enhancing Se biofortification, leveraging the interactive mechanisms of Se–Cd antagonism in the soil–rice system. Based on the results of pot experiments, this study employed a uniform design method to optimize the formulation ratios of four materials. The results showed that soil conditioners have a significant positive impact on rice growth in Cd-contaminated soil. The application of soil conditioners could effectively reduce the available Cd concentration in slightly Cd-contaminated rice soils in a Se-enriched area of Jiangxi Province and regulate the enrichment coefficient and the transport coefficient of soil Cd and Se in various organs of rice. The theoretically optimal formulation predicted by the regression model was: lime, 1,500 kg hm^−2^; Ca–Mg–P fertilizer, 7,500 kg hm^−2^; and sodium humate, 870 kg hm^−2^. The experiment found that the T3 treatment was the most effective at inhibiting Cd accumulation and promoting Se accumulation. The formulation for the T3 treatment was: lime, 750 kg hm^−2^; Ca–Mg–P fertilizer, 6,000 kg hm^−2^; sodium humate, 4,500 kg hm^−2^; and zeolite, 0 kg hm^−2^. The application of T3 soil conditioner maximizes the reduction of Cd and increases the concentration of Se in rice grains, significantly enhancing rice yield. With the application of T3 soil conditioner, the estimate of the average soil available Cd concentration was 0.13 mg/kg. Compared with the control, the T3 soil conditioner reduced the available Cd concentrations by 43.4%. In addition, the T3 soil conditioner increased the available Se concentration by 0.0192 mg/kg compared with the control group. This enables high-yield cultivation of rice in Cd-polluted farmland with Se-enriched soil.

## 1 Introduction

Selenium (Se) is one of the essential trace nutrients for human survival (Templeton and Liu, 2010). It plays a vital role in numerous physiological processes within the human body, exhibiting multiple benefits such as antioxidant properties, anticancer effects, and immune system enhancement (Fordyce, 2012). In China, the daily Se intake among national average per capita adults is only 26.63 μg, far below the 60–240 μg recommended by the Chinese Nutrition Society (Dinh et al., 2018). As the primary staple crop, rice is the main source of Se intake for residents. Therefore, the cultivation of Se-enriched rice in high-Se condition regions to increase residents’ Se intake by boosting the Se concentration in rice grains is a viable approach (Wang et al., 2013).

Natural Se-enriched regions in China typically exhibit high levels of heavy metals in their geological background, with cadmium (Cd) being the most common associated metal (Chang et al., 2020). Cd is a chronic potent nephrotoxic agent classified as a class 1 human carcinogen by the International Agency for Research on Cancer (Guo et al., 2024). Rice is the major dietary source of Cd exposure, particularly in East and Southeast Asia where rice is the staple cereal food. For instance, during the period from 1998 to 2001, rice consumption accounted for 36%–50% of the total dietary Cd exposure in the Japanese general population (Kikuchi et al., 2008). A Chinese survey indicated that heavy metals and metalloids account for 82.4% of contaminated soil sites (Yang et al., 2018). Among these heavy metals and metalloids, Cd had the highest rate of exceeding the permissible levels in soil samples. Soil Cd is absorbed by plant roots, which is then transferred to the plant’s aboveground parts and accumulates in the edible sections, ultimately entering the human body through consumption. Cd and Se exhibit a certain degree of antagonism; however, the Cd levels in rice grains still exceed standards in naturally Se-enriched regions (Guo et al., 2021). Therefore, achieving low Cd levels in rice grains in Se-enriched areas remains a formidable challenge. Numerous studies have shown that low Se concentrations promoted plant growth and development, regulated the absorption and transport of Cd in crops, and antagonized heavy metal ions, thereby mitigating the harmful effects of Cd on plants (Zhu et al., 2024a). Se can mitigate the toxicity of Cd by regulating the plant’s antioxidant system and eliminating Cd-induced excess reactive oxygen species (ROS). A number of studies found that the application of Se reduced the surface area of plant roots, thereby decreasing the roots’ uptake of heavy metals (Ismael et al., 2019).

Currently, the addition of external Se is a commonly used method to inhibit and control Cd accumulation. Se supplementation has shown great potential to limit the uptake and transport of Cd in rice (Wang et al., 2026). There is currently a wide variety of soil conditioners available on the Chinese market containing a diverse range of active ingredients such as silicon (Si), Se, zinc (Zn), organic fertilizers, and lime. However, the majority of soil conditioners consist of a single component, and the results of their application have been inconsistent, leading to poor suitability (Zhang et al., 2026). A study found that, under conditions of moderate to mild Cd contamination, the application of Se fertilizer partially alleviated the heavy metal toxicity to plants and reduced the plant uptake of Cd from the soil, thereby effectively lowering Cd accumulation in rice grains (Huang et al., 2021). However, Se fertilizers are relatively expensive, and their application is significantly affected by climate conditions (Demin et al., 2022). The effectiveness of Se fertilizer varies significantly under different Cd contamination conditions (Lin et al., 2012). Although a number of studies have investigated the effects of Se fertilizer on Cd accumulation at the laboratory and field levels, research on low-cost and stable Se-enriched and Cd-reduced conditioners has not yet been conducted (Arinzechi et al., 2024).

The present study was conducted using indoor potted plants and field experiments to investigate the potential role of Se-enriched and Cd-reduced conditioners in the uptake/translocation of Cd and the growth of rice plants. We aimed to provide a basis for the development of strategies to reduce the risks associated with Cd toxicity and maintain sustainable plant production.

## 2 Materials and methods

### 2.1 Study site description

The study sites are geographically located at Jieguan town, Shanggao county, Yichun City, northwest Jiangxi Province, Central China (28°07′ N, 115°10′ E). This region features a subtropical monsoon climate with an average annual temperature of 17.6°C, accumulated temperature ≥10°C of 5,400°C, and average annual precipitation of 1,718.4 mm. Precipitation from April to June averages 763.6 mm, accounting for 44% of the annual total. The frost-free period lasts approximately 276 days, with average annual sunshine hours of 1,668.2 h. Yichun City is one of China’s three major naturally Se-rich regions (Se content >0.4 mg/kg), with Se-enriched soil covering an area of 5,200 km^2^. Soil Se mainly originates from quaternary river sediments. The soil is classified as red soil in the Chinese Soil Taxonomy, which is equivalent to Acrisols in the World Reference Base for Soil Resources. The soil for the pot plant experiment was taken from Jieguan town.

### 2.2 Plant material and experimental design

The rice variety used in both pot experiments and field trials was Yongyou 1538. Rice was transplanted on 20 July 2024 and was harvested on 31 October 2024, for a total of 103 days. The test rice varieties are the main cultivated varieties suitable for local planting.

For the potted plant experiment, five treatment groups were established, each with three replicates. Each pot was filled with 10 kg soil and planted with three rice seedlings. The treatments included lime, Ca–Mg–P fertilizer, zeolite, and sodium humate as soil conditioners, with the control group (CK) receiving no conditioner application.

For the field experiment, four factors (i.e., lime, Ca–Mg–P fertilizer, zeolite, and sodium humate) were used as uniformly designed experimental factors. Each factor had nine levels, employing a four-factor, nine-level uniform design table, U_9_(9^4^), to set up nine experimental treatments (**Table 1**). CK treatment without any conditioning agent was also established, resulting in a total of 10 treatments. Detailed experimental procedures and the material sources are referenced in the **Supplementary Material**.

**Table 1:** Uniform design table of U_9_(9^4^).

Treatment	Lime, *X*_1_	Ca–Mg–P fertilizer, *X*_2_	Zeolite, *X*_3_	Sodium humate, *X*_4_
CK	0	0	0	0
T1	1 (0)	3 (1,500)	7 (4,500)	9 (6,000)
T2	2 (375)	6 (3,750)	4 (2,250)	8 (5,250)
T3	3 (750)	9 (6,000)	1 (0)	7 (4,500)
T4	4 (1,125)	2 (750)	8 (5,250)	6 (3,750)
T5	5 (1,500)	5 (3,000)	5 (3,000)	5 (3,000)
T6	6 (1,875)	8 (5,250)	2 (750)	4 (2,250)
T7	7 (2,250)	1 (0)	9 (6,000)	3 (1,500)
T8	8 (2,625)	4 (2,250)	6 (3,750)	2 (750)
T9	9 (3,000)	7 (4,500)	3 (1,500)	1 (0)

Numbers outside parentheses are the codes in the uniform design table, while the values in parentheses are the dosage levels in kilograms per square hectometer.

*CK*, control group.

### 2.3 Sample collection and determination methods

Rice plant samples were collected at maturity. The pot plant experiment collected all rice plants and soil. Plants were collected using a five-point sampling method in the field trial. Five rice plant samples were collected from the plot. We used a soil auger to collect soil samples. At each plot, 20 random samples were taken near the rice plants (depth, 20 cm), and all the collected soil was thoroughly mixed to form a composite soil sample. Rice plants were separated into root, stem, leaf, and grain. The four parts were successively cleaned with tap water and deionized water, the root, stem, and leaf at 105°C under 30 min, and then cooling to 40°C drying to constant weight. Grain was roasted after natural air drying, and all plant samples were crushed, sieved, and placed in dry self-sealing bags for spare parts. Three rice plants were selected from each plot for variety identification.

The soil pH and electrical conductivity (EC) were measured in a suspension with deionized water (soil-to-water ratio of 1:2.5) using a pH meter (FE-20K; METTLER TOLEDO, Columbus, OH, USA) and a conductivity meter (DDSJ-308F; INESA, Shanghai, China), respectively. Soil organic matter (SOM) was analyzed using wet digestion with K_2_Cr_2_O_7_–H_2_SO_4_. Total nitrogen (TN) was determined with the semi-micro Kjeldahl method, and alkaline-N was determined with the alkaline diffusion method. Available P was determined using the 0.05 mol L^−1^ HCl–0.025 mol L^−1^ (1/2H_2_SO_4_) method, while available K was leached with 1 mol L^−1^ NH_4_OAC and determined using the flame photometric method. Dried root, leaf, stem, root, and soil samples were ground and digested in a mixture of HNO_3_–HClO_4_ (4:1). For the extraction of available Cd and available Se from the soil and plant organs, as well as the total soil Cd, refer to the **Supplementary Material**. The concentrations of Cd and Se were determined using inductively coupled plasma atomic emission spectroscopy (ICP-OES) (PerkinElmer 8300, Shelton, CT, USA), which followed the method of Zhang et al. (2019).


Fruiting rate=Number of solid grains/Total number of grains×100%

$$\text{Theoretical yield}\left(kg/hm^{2}\right)=(\text{Number of effective spikes}\times \text{Total number of grains per spikes}\times \text{Fruiting rate}\times \text{Thousand grain weight})/(1,000\times 1,000)$$

The transfer factor (TF) from organ “a” to organ “b” was calculated using the following formula (Li et al., 2016): TF a–b = Cd concentration in organ “b” (mg/kg)/Cd concentration in organ “a” (in milligrams per kilogram).

### 2.4 Statistical analyses

Experimental data were analyzed using Excel 2019. Analysis of variance (ANOVA) and Duncan’s method were performed using SPSS 22.0 to estimate differences in the different treatments (*p* < 0.05). Origin 2021 was used for generating graphs.

## 3 Results

### 3.1 Effect of a single material on soil physicochemical properties and plant growth and development

**Figure 1:**
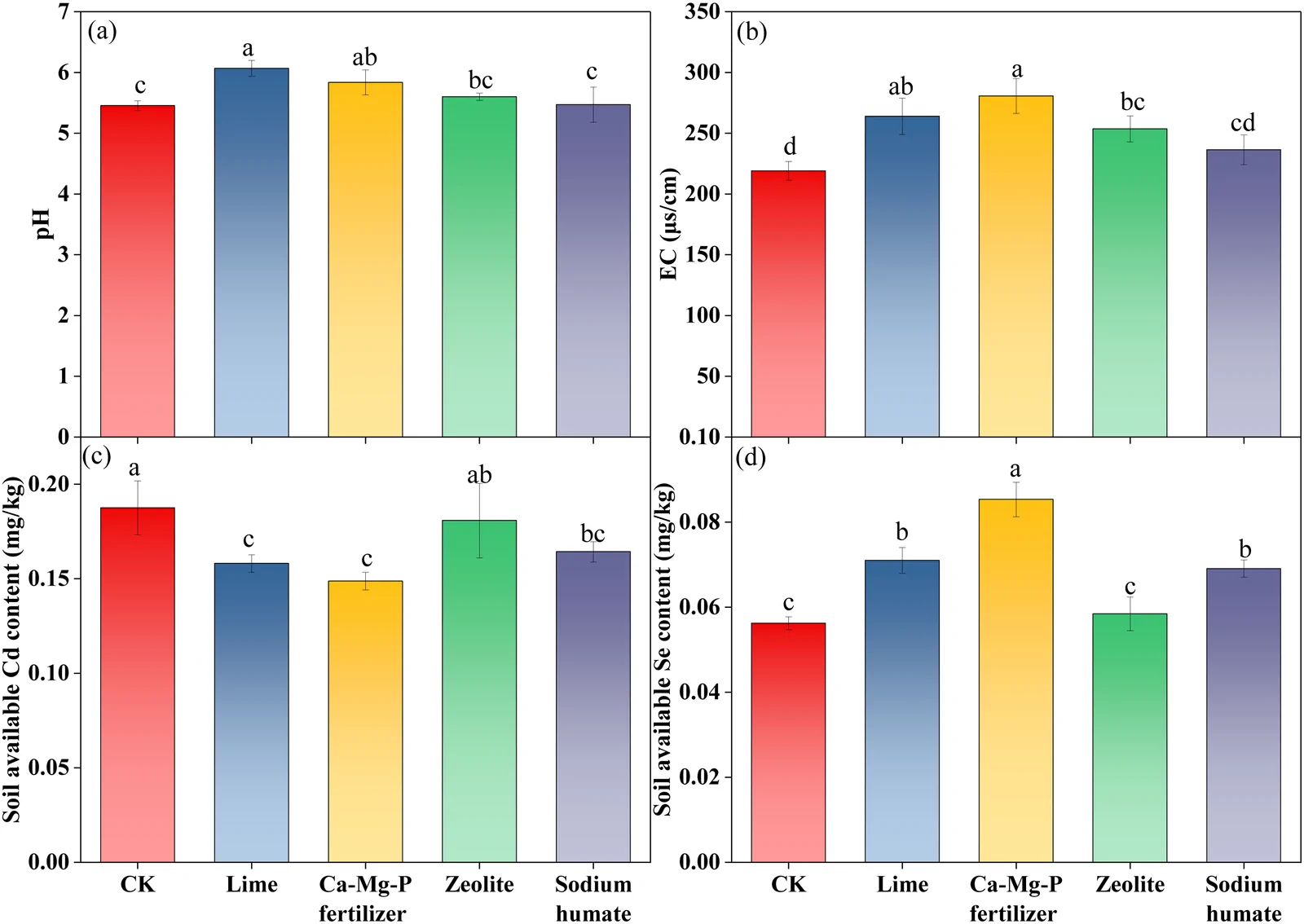
Effect of a single material on the physicochemical properties of soil (*n* = 3). Significant difference (*p* < 0.05) is denoted by *different letters*. *Columns* and *bars* indicate the mean and standard deviation. *CK*, control. pH **(a)**; EC **(b)**; Soil available Cd concentration **(c)**; Soil available Se concentration **(d)**.

The treatment and control group soils were acidic, with pH values ranging from 5.45 to 6.07 (**Figure 1A**). The soil EC of the treatment group averaged 258.6 μS/cm, which was higher than that in the control group (219.0 μS/cm) (**Figure 1B**). This indicates that the application of a single material increases the soil pH and EC. As shown in **Figures 1C, D**, the different single materials had significant effects on the concentrations of Cd and Se. For the potted plant experiment, the lime, Ca–Mg–P fertilizer, and sodium humate treatments significantly decreased the Cd concentration compared with the control group (*p* < 0.05). The lime, Ca–Mg–P fertilizer, and sodium humate treatments significantly increased the available Se concentration compared with the control group (*p* < 0.05), with the increase ranging from 22.95% to 51.78%. Zeolite treatment had no effect on the soil Se concentration (*p* > 0.05).

The lime, Ca–Mg–P fertilizer, and sodium humate treatments significantly increased the fruiting rate and solid grains of spike compared with the control group (*p* < 0.05, **Table 2**). The solid grains and theoretical yield of rice under the lime and Ca–Mg–P fertilizer treatments were significantly higher than that under zeolite, sodium humate, and control treatments (*p* < 0.05). The actual yield of the group with single material treatment was higher than that of the control group; however, there was no significant difference between the treatment group and the control group (*p* > 0.05).

**Table 2:** Effect of a single material on plant yield and its constituent factors.

Seed parameters	CK	Lime	Ca–Mg–P fertilizer	Zeolite	Sodium humate
Plant height (cm)	98.33 ± 3.21a	104.33 ± 3.06a	103.67 ± 2.31a	99.00 ± 5.29a	103.33 ± 4.51a
Stem weight (g/pot)	47.58 ± 3.82c	56.37 ± 3.90bc	66.33 ± 8.40a	61.13 ± 3.52ab	52.00 ± 4.72bc
Effective spikes (spikes/pot)	42.00 ± 2.65ab	35.67 ± 3.51bc	33.00 ± 2.65c	42.33 ± 4.16a	33.33 ± 4.16c
Empty grains (1,000 grains/pot)	2.23 ± 0.09a	1.32 ± 0.03d	1.26 ± 0.09d	1.95 ± 0.10b	1.72 ± 0.04c
Solid grains (1,000 grains/pot)	3.69 ± 0.16b	4.34 ± 0.23a	4.38 ± 0.18a	3.76 ± 0.22b	3.56 ± 0.24b
Solid grains of spike	88.20 ± 8.82c	122.08 ± 6.53ab	132.97 ± 6.36a	89.55 ± 12.94c	107.68 ± 12.32b
Fruiting rate (%)	62.32 ± 1.58c	76.62 ± 1.33a	77.62 ± 0.88a	65.76 ± 2.06b	67.43 ± 1.88b
Thousand grain weight (g)	18.66 ± 0.13c	20.07 ± 0.39a	19.56 ± 0.56ab	19.02 ± 0.20bc	19.74 ± 0.68ab
Theoretical yield (g/pot)	68.85 ± 3.04b	87.14 ± 6.02a	85.60 ± 3.74a	71.43 ± 3.63b	70.27 ± 4.83b
Actual yield (g/pot)	69.62 ± 7.81a	82.52 ± 6.56a	78.91 ± 5.82a	71.55 ± 1.68a	70.21 ± 15.29a

Different lowercase letters in the same column indicate significant differences between different treatments under the same variety (*p* < 0.05). Values are the mean ± SE.

*CK*, control group.

### 3.2 Effect of complex soil conditioners on soil physicochemical properties and plant growth and development

Among nine soil conditioners, T9 was effective in increasing the soil pH (*p* < 0.05) (**Table 3**). Among nine soil conditioners, T9 was effective in increasing the soil pH (p < 0.05;table 3) Compared with the CK, the T3, T8, and T9 treatments had the largest increase in soil EC (*p* < 0.05). However, the EC values also increased in the other treatments, but with no significant difference among treatments (*p* > 0.05). T1 and T2 treatments had the highest organic matter concentration (*p* < 0.05), while the other treatments showed no significant difference from the control (*p* > 0.05). Soil TN was not significantly affected by the different conditioner treatments. The available phosphorus (AP) concentration on T3 was significantly higher than that of the other soil conditioners and the control (*p* < 0.05). The highest available potassium (AK) concentration was found on T8 (*p* < 0.05). In summary, T3 and T9 were the most effective in improving the soil physicochemical properties.

**Table 3:** Effects of composite soil conditioners on the soil physicochemical properties.

Treatment	pH	EC (μS/cm)	SOM (g/kg)	TN (g/kg)	AP (mg/kg)	AK (mg/kg)
CK	5.4 ± 0.29c	132.85 ± 10.5cd	35.72 ± 1.12b	2.04 ± 0.09a	25.73 ± 4.17bc	178.57 ± 7.14c
T1	5.82 ± 0.09b	135.29 ± 2.59cd	38.89 ± 1.63a	2.03 ± 0.04a	21.05 ± 3.57c	219.05 ± 22.96abc
T2	5.99 ± 0.11ab	141.98 ± 8.65c	38.70 ± 1.67a	2.02 ± 0.06a	26.69 ± 4.41abc	240.48 ± 29.74ab
T3	5.94 ± 0.48ab	189.15 ± 3.46a	36.26 ± 0.19b	2.01 ± 0.05a	33.14 ± 1.29a	245.24 ± 32.71ab
T4	5.91 ± 0.19ab	125.15 ± 8.63d	35.91 ± 0.40b	2.03 ± 0.05a	20.76 ± 3.68c	188.1 ± 39.34c
T5	6.03 ± 0.30ab	142.36 ± 8.74c	35.81 ± 0.15b	1.99 ± 0.05a	27.62 ± 1.11ab	197.62 ± 35.95bc
T6	6.00 ± 0.21ab	140.21 ± 4.16c	35.81 ± 0.86b	2.02 ± 0.08a	28.23 ± 4.36ab	207.14 ± 25.75abc
T7	5.88 ± 0.18b	123.72 ± 6.95d	35.45 ± 1.90b	2.01 ± 0.08a	24.16 ± 1.93bc	173.81 ± 33.76c
T8	5.90 ± 0.17ab	168.14 ± 8.79b	34.96 ± 1.18b	2.03 ± 0.06a	29.87 ± 4.86ab	252.38 ± 9.51a
T9	6.41 ± 0.35a	184.21 ± 5.66a	34.57 ± 1.98b	1.97 ± 0.10a	30.83 ± 3.02ab	214.28 ± 12.37abc

Different lowercase letters in the same column indicate significant differences between the different treatments under the same variety (*p* < 0.05). Values are the mean ± SE.

EC, electrical conductivity; SOM, soil organic matter; TN, total nitrogen; AP, available phosphorus; AK, available potassium.

There were no significant differences between the treatment groups and the control group in terms of empty grains, fruiting rate, and thousand grain weight (*p* > 0.05) (**Table 4**). There were no significant differences between the treatment groups and the control group in terms of empty grains, fruiting rate, and thousand grain weight (p > 0.05; table 4). T5 and T9 had the highest effective spikes and solid grains (*p* < 0.05), while the other treatments showed no significant difference from the control (*p* > 0.05). Compared with the control group, the treatment groups had higher theoretical yield and actual yield. The results showed that the theoretical yield and the actual yield under the T5 and T9 treatments were significantly higher than those of the other treatment groups and the control group (*p* < 0.05).

**Table 4:** Effect of composite soil conditioners on plant yield and its constituent factors.

Treatment	Effective spikes (spikes/3 rice plants)	Empty grains (1,000 grains/3 rice plants)	Solid grains (1,000grains/3 rice plants)	Fruiting rate (%)	Thousand grain weight (g)	Theoretical yield (kg/hm^2^)	Actual yield (kg/hm^2^)
CK	46.00 ± 4.00b	2.27 ± 0.49a	6.45 ± 0.38cd	74.09 ± 3.21ab	21.74 ± 0.48a	7,572.1 ± 281.57b	7,294.44 ± 98.53cd
T1	49.00 ± 1.00ab	1.94 ± 0.29a	6.63 ± 0.39bcd	77.39 ± 3.64ab	21.55 ± 0.75a	7,719.22 ± 498.61b	7,923.24 ± 244.11ab
T2	48.00 ± 1.00ab	2.44 ± 0.37a	6.45 ± 0.31cd	72.66 ± 2.51ab	21.76 ± 0.31a	7,584.57 ± 430.51b	7,518.44 ± 230.07bcd
T3	49.67 ± 3.21ab	2.23 ± 0.32a	6.78 ± 0.26abcd	75.37 ± 2.31ab	21.79 ± 0.43a	7,978.49 ± 301.27ab	7,838.44 ± 196.45ab
T4	47.67 ± 1.15ab	2.52 ± 0.54a	6.49 ± 0.36d	72.12 ± 5.07b	21.98 ± 0.72a	7,695.50 ± 398.04b	7,668.84 ± 390.00bc
T5	50.33 ± 1.15a	1.94 ± 0.67a	7.36 ± 0.36a	79.24 ± 6.34a	21.36 ± 0.45a	8,495.09 ± 585.70a	8,209.64 ± 309.64a
T6	49.33 ± 0.58ab	1.91 ± 0.16a	6.73 ± 0.49abcd	77.88 ± 1.42ab	21.78 ± 0.12a	7,911.64 ± 540.44ab	7,312.04 ± 281.21cd
T7	48.00 ± 1.73ab	1.97 ± 0.26a	6.38 ± 0.13d	76.41 ± 2.61ab	21.57 ± 0.32a	7,435.04 ± 149.61b	7,040.04 ± 408.04d
T8	49.00 ± 1.00ab	2.21 ± 0.28a	7.08 ± 0.49abc	76.17 ± 2.41ab	21.28 ± 0.35a	8,126.06 ± 439.00ab	7,872.04 ± 115.30ab
T9	51.00 ± 1.00a	1.98 ± 0.35a	7.22 ± 0.04ab	78.51 ± 2.90ab	21.89 ± 0.15a	8,531.53 ± 54.07a	8,318.44 ± 125.08a

Different lowercase letters in the same column indicate significant differences between different treatments under the same variety (*p* < 0.05). Values are the mean ± SE.

*CK*, control group.

### 3.3 Accumulation and transportation of Se and Cd in the soil–rice system

**Figure 2:**
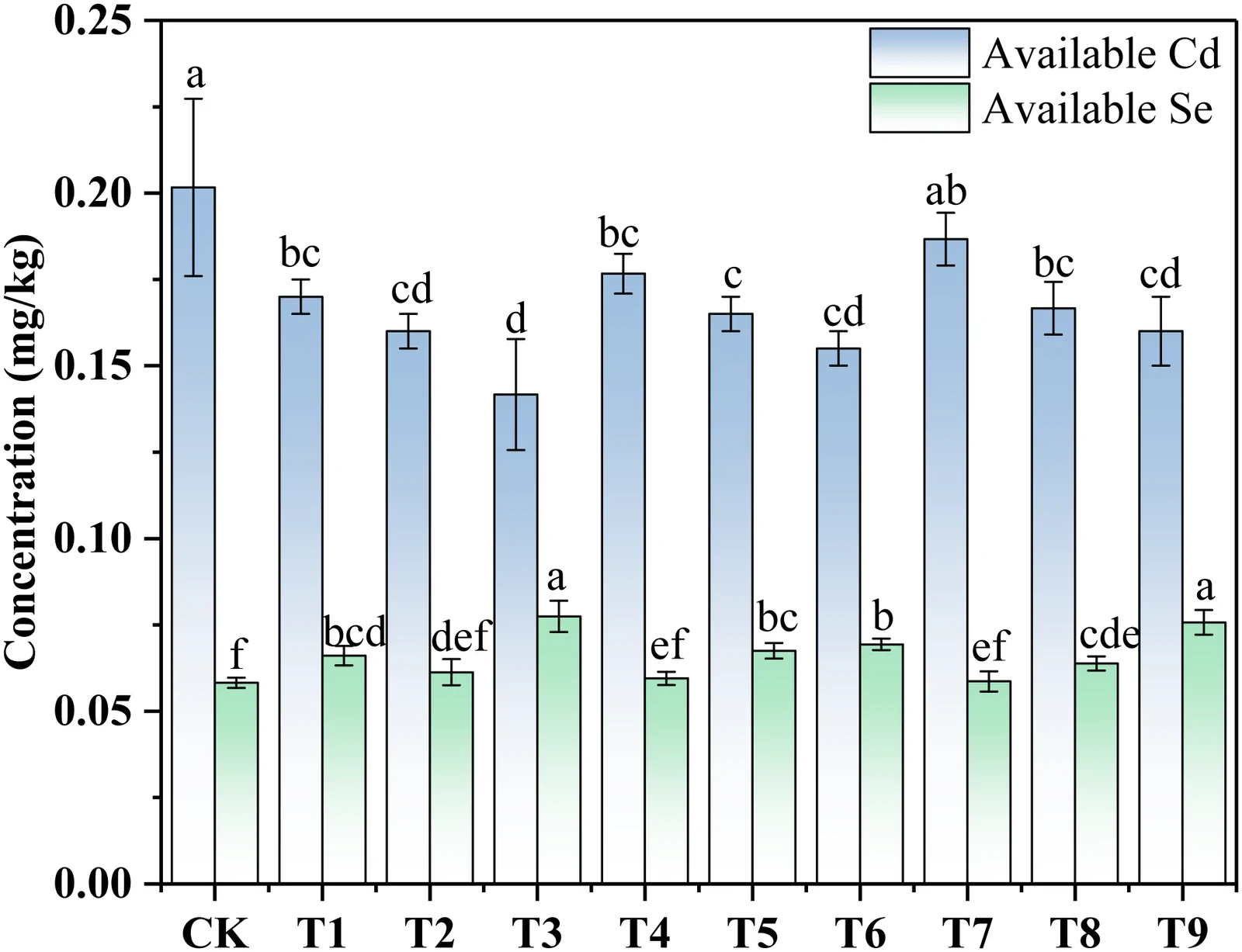
Effects of composite soil conditioners on the available cadmium (Cd) and selenium (Se) concentrations in soil (*n* = 3). Significant difference (*p* < 0.05) is denoted by *different letters*. *Columns* and *bars* indicate the mean and standard deviation. *CK*, control.

The available Cd concentration in the soil ranged from 0.13 to 0.23 mg/kg, with a coefficient of variation (CV) of 11.15%, indicating high spatial heterogeneity. Compared with the control, the treatments significantly reduced the soil available Cd concentration, with the T3 treatment showing the most effective Cd reduction. Overall, the concentration of available Se was significantly lower than that of available Cd, and the T3 and T9 treatments were the most effective for the enrichment of available See (**Figure 2**).

The Cd concentration was higher in the root than in the aboveground organs of rice. In the control and treatment groups, the average concentration of Cd in the root was 6.45 mg/kg, with a range from 4.79 (T3) to 9.13 mg/kg (CK); in the stem was 3.76 mg/kg, with a range from 2.98 (T6) to 5.79 mg/kg (CK); in the leaf was 1.93 mg/kg, with a range from 1.59 (T3) to 2.31 mg/kg (CK); and in the seed was 0.80 mg/kg, with a range from 0.63 (T3) to 1.28 mg/kg (CK). The order of average Cd concentration was as follows: root > stem > leaf > seed.

**Figure 3:**
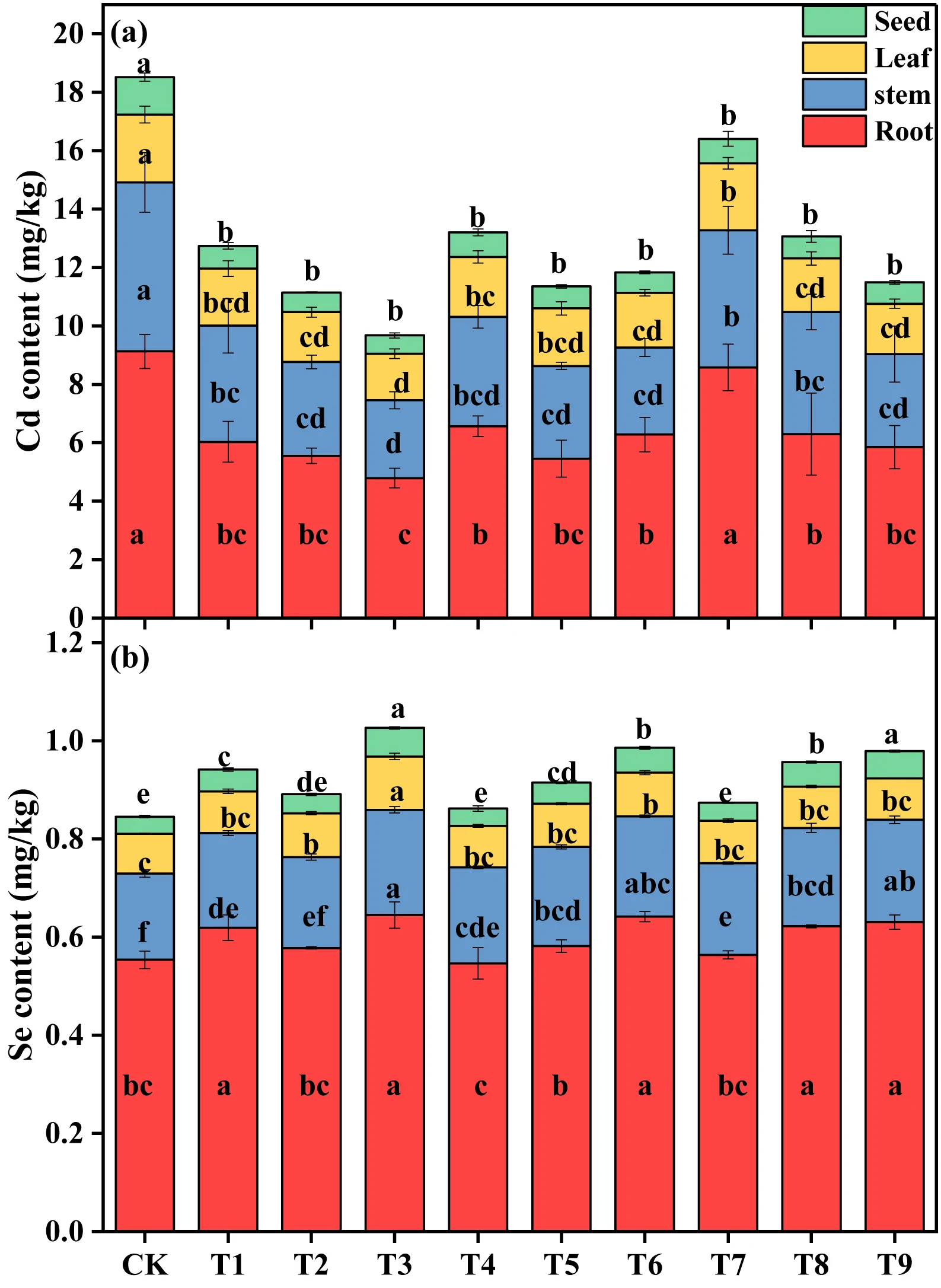
Distribution of cadmium (Cd) and selenium (Se) in each organ of rice in different soil conditioners (*n* = 3). Significant difference (*p* < 0.05) is denoted by *different letters*. *Columns* and *bars* indicate the mean and standard deviations. *CK*, control.

The Se concentration was higher in the root than in the aboveground organs of rice (**Figure 3**). In the control and treatment groups, the average concentration of Se in the root was 0.60 mg/kg, with a range from 0.55 (CK) to 0.64 mg/kg (T3); in the stem was 0.20 mg/kg, with a range from 0.18 (CK) to 0.21 mg/kg (T3); in the leaf was 0.09 mg/kg, with a range from 0.081 (CK) to 0.11 mg/kg (T3); and in the seed was 0.04 mg/kg, with a range from 0.034 (CK) to 0.058 mg/kg (T3). The order of average Se concentration was as follows: root > stem > leaf > seed.

The Cd and Se accumulation ability of the different soil conditioners was significantly different. The Cd concentration of the root of T3 was 0.52 times that of CK (*p* < 0.05), and the Se concentration of the root of T3 was 1.16 times that of CK (*p* < 0.05). The variation in the Cd and Se concentrations in other rice organs was similar to that in the root system.

### 3.4 Transfer factor of Se and Cd between various organs

TF was used to measure the Cd and Se transport and redistribution ability between different organs (**Table 5**). TF was used to measure the Cd and Se transport and redistribution ability between different organs (table 5). All organs were chelated a portion of Cd and reduced Cd transport (TF < 1). Nevertheless, there were differences in the chelating ability, with the roots exhibiting the strongest Cd chelating ability. There were significant differences in the Cd TFs of the different soil conditioners, particularly the leaf–seed TF. The maximum TF value of leaf–seed was 1.45 times higher than the minimum value. The leaf–seed TF and the stem–leaf TF were lower than the root–stem TF.

**Table 5:** Effects of soil conditioners on the cadmium (Cd) and selenium (Se) transport coefficients of rice.

Treatment	Cd			Se		
	Root–stem TF	Stem–leaf TF	Leaf–seed TF	Root–stem TF	Stem–leaf TF	Leaf–seed TF
CK	0.68 ± 0.09a	0.60 ± 0.09a	0.54 ± 0.12a	0.32 ± 0.01c	0.46 ± 0.03abc	0.43 ± 0.03de
T1	0.66 ± 0.10ab	0.52 ± 0.10ab	0.40 ± 0.08a	0.31 ± 0.01c	0.44 ± 0.03bcd	0.52 ± 0.02bc
T2	0.58 ± 0.05ab	0.53 ± 0.05ab	0.40 ± 0.03a	0.32 ± 0.02c	0.48 ± 0.03ab	0.43 ± 0.04de
T3	0.56 ± 0.10ab	0.60 ± 0.10a	0.40 ± 0.09a	0.33 ± 0.01bc	0.51 ± 0.02a	0.53 ± 0.02bc
T4	0.57 ± 0.05ab	0.55 ± 0.05ab	0.42 ± 0.08a	0.36 ± 0.02a	0.43 ± 0.02cd	0.42 ± 0.08e
T5	0.59 ± 0.09ab	0.62 ± 0.09a	0.39 ± 0.08a	0.35 ± 0.01ab	0.43 ± 0.01cd	0.49 ± 0.02cd
T6	0.48 ± 0.06b	0.64 ± 0.06a	0.37 ± 0.04a	0.32 ± 0.01c	0.44 ± 0.02cd	0.57 ± 0.04b
T7	0.54 ± 0.05ab	0.50 ± 0.05b	0.37 ± 0.13a	0.33 ± 0.02bc	0.46 ± 0.01abc	0.42 ± 0.03e
T8	0.57 ± 0.06ab	0.44 ± 0.06b	0.41 ± 0.08a	0.32 ± 0.01c	0.42 ± 0.03cd	0.59 ± 0.04b
T9	0.58 ± 0.19ab	0.58 ± 0.13ab	0.43 ± 0.07a	0.33 ± 0.01bc	0.41 ± 0.01d	0.66 ± 0.02a

Different lowercase letters in the same column indicate significant differences between different treatments under the same variety (*p* < 0.05). Values are the mean ± SE.

CK, control group; TF, transfer factor.

The Se root–stem TF ranged from 0.31 to 0.36, the stem–leaf TF ranged from 0.41 to 0.51, and the leaf–seed TF ranged from 0.42 to 0.66. T4 had the highest Se root–stem transport capacity, while T3 had the highest Se stem–leaf transport capacity. T9 had the highest Se leaf–seed transport capacity. The leaf–seed and stem–leaf TFs were higher than the root–stem TF.

### 3.5 Correlation analysis of the Cd and Se concentrations in various organs

**Figure 4:**
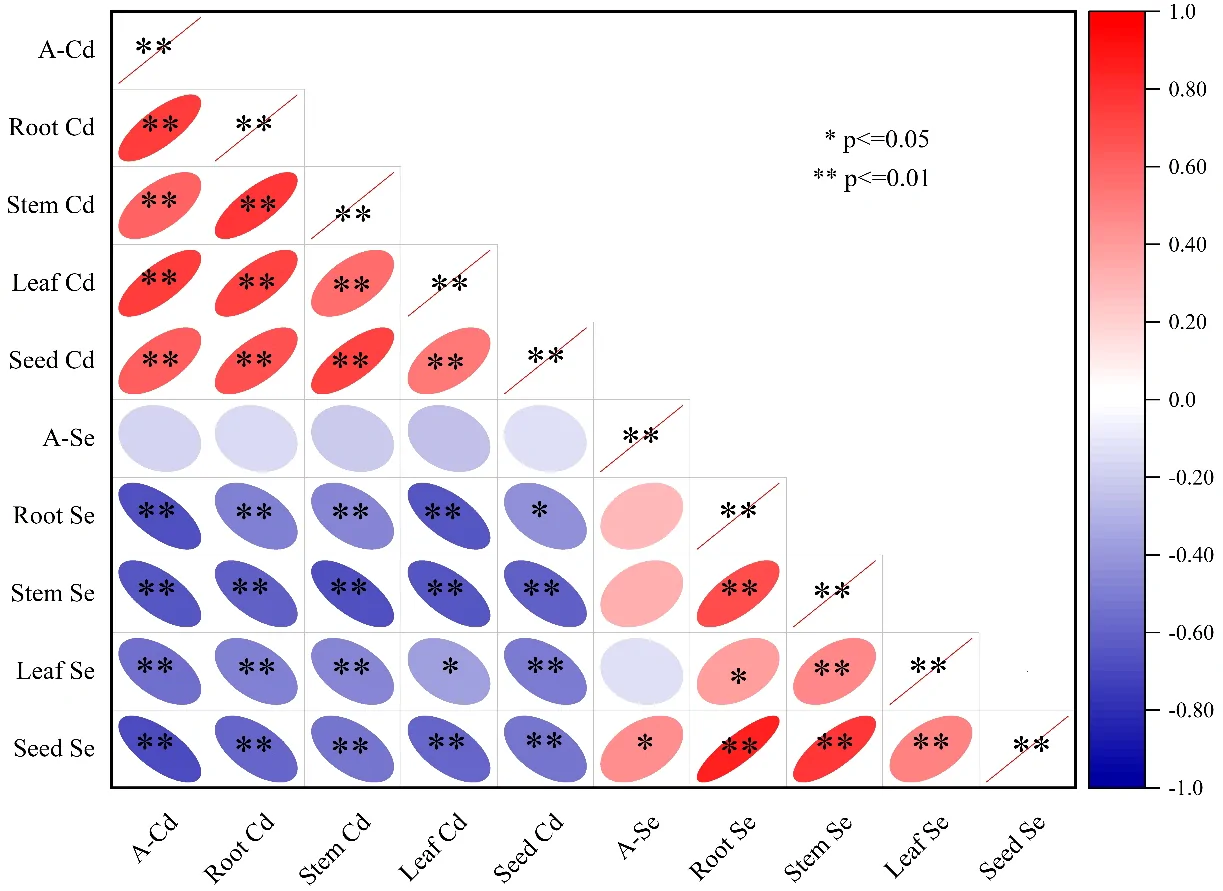
Correlation between the cadmium (Cd) and selenium (Se) concentrations in rice organs (*n* = 30). *Red ovals* indicate a positive correlation, while *blue ovals* indicate a negative correlation. *p ≤ 0.05, **p ≤ 0.01. *A-Se*, available selenium; *A-Cd*, available cadmium.

The Cd concentrations of the rice organs were positively correlated, and the correlation reached a highly significant level (*p* < 0.01) (**Figure 4**). The Cd concentrations of the root and stem showed the strongest correlation, with a correlation coefficient of 0.766. The Se concentrations of the different organs were positively correlated. The Se concentrations of the root and seed showed the strongest correlation, with a correlation coefficient of 0.856. The Cd concentrations of the root, stem, leaf, and seed were significantly negatively correlated with the Se concentrations of the root, stem, leaf, and seed.

### 3.6 Correlation between soil physicochemical properties and available Cd and Se

**Figure 5:**
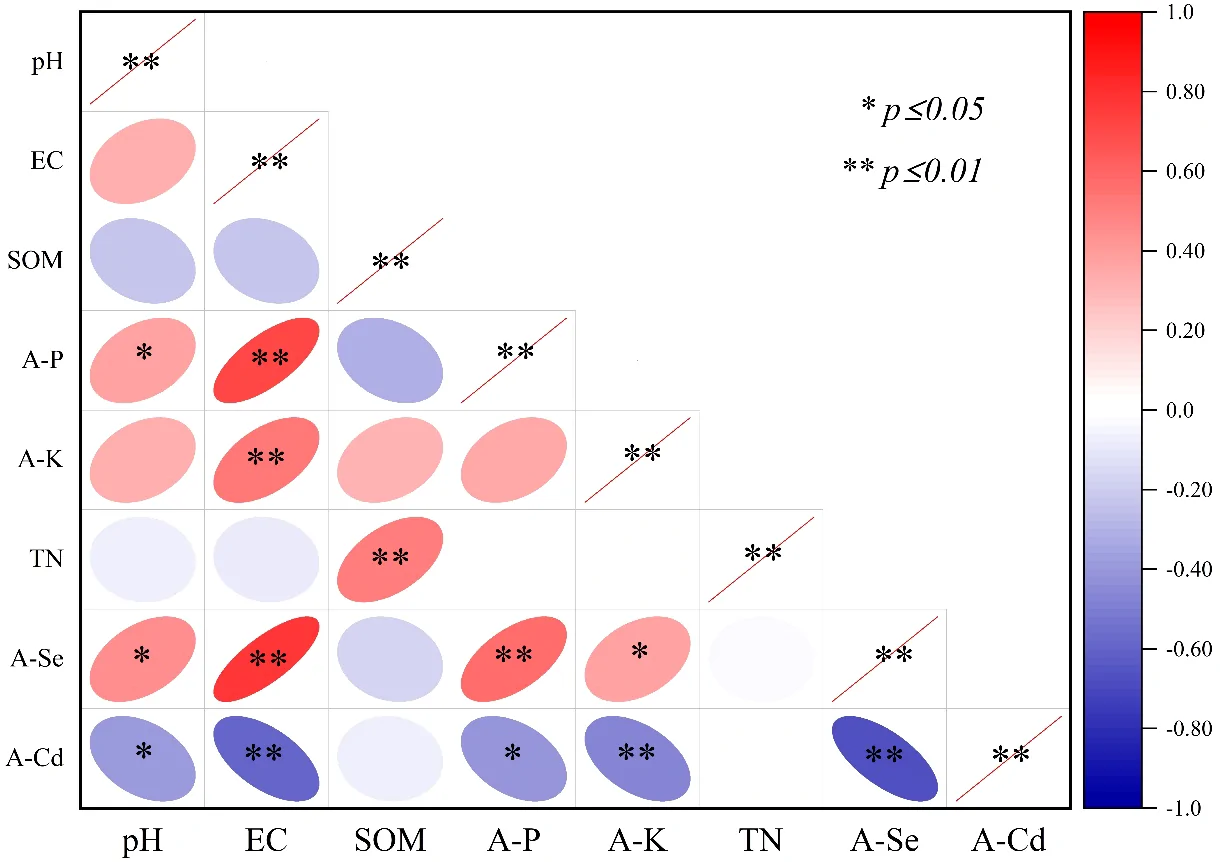
Correlation between the soil physicochemical properties and available cadmium (Cd) and selenium (Se) (*n* = 30). *Red ovals* indicate a positive correlation, while *blue ovals* indicate a negative correlation. *p ≤ 0.05, **p ≤ 0.01. *EC*, electrical conductivity; *SOM*, soil organic matter; *A-P*, available phosphorus; *A-K*, available potassium; *TN*, total nitrogen; *A-Se*, available selenium; *A-Cd*, available cadmium.

To further explore the interactions between the soil physicochemical properties (i.e., pH, EC, SOM, AP, AK, and TN) and the available Cd and Se, correlation analyses were performed Fig. 5. Available Cd showed negative correlations with the physicochemical properties (**Figure 5**). Available Se showed positive correlations with the physicochemical properties. Soil pH was negatively correlated with available Cd, but was positively correlated with available Se and AP. There was a positive correlation between TN and SOM (*p* < 0.01). EC was positively correlated with AP, AK, and available Se (*p* < 0.01), but was negatively correlated with available Cd (*p* < 0.01).

### 3.7 Use of a multiple regression model to calculate the optimal soil conditioner formulation

Based on the formulation of the soil conditioners and the experimental results on Cd content in late-season rice grains, a multivariate linear stepwise regression analysis was performed using the experimental data. The equations describing the relationship between the Cd reduction rate in grains under different soil conditioner treatments and the various factors are as follows:


$$Y\;=\;66.13-0.17*X_{1}+0.038*X_{2}-0.075*X_{4}\left(Fvalue:27.6375,R^{2}\;=\;0.943119,p\;=\;0.0015\right)$$

The optimized formulation, determined through calculation, was as follows: lime, 1,500 kg hm^−2^; Ca–Mg–P fertilizer, 7,500 kg hm^−2^; and sodium humate, 870 kg hm^−2^. The ratio of limestone to Ca–Mg–P fertilizer to sodium humate was 1:5:0.58. The regression equation for late-season rice was statistically significant. The error between the observed and the fitted values was very small, indicating high accuracy, as shown in **Supplementary Table 2**.

## 4 Discussion

### 4.1 Effect of soil conditioners on the Cd and Se concentrations in soil

The concentrations of available Cd and available Se in soil are typical key indicators reflecting their biological availability (Kunene et al., 2020). An increased soil pH enhances the adsorption capacity of soil colloids and clay particles for heavy metal ions, promoting the transformation of Cd from an unstable to a stable state, thereby reducing the availability and mobility of soil Cd (Soriano-Disla et al., 2010). In this study, the application of soil conditioners significantly increased the soil pH, and the available Cd concentrations were significantly lower than those in the CK. The soil conditioners had not only physical adsorption but also chemical adsorption of Cd, primarily occurring on the surfaces of mineral materials. Chemical adsorption primarily involves the formation of chelates between Si–Ca–Mg ions and Cd, further reducing the available Cd concentration in the soil. This also verified that T3 has better effectiveness in reducing the available Cd concentration (Wang et al., 2004).

In contrast to Cd, Se was more active in alkaline soils. The Cd in soil typically exists as a cation with a tendency to lose electrons, while Se primarily occurs as selenate or selenite anions with a tendency to gain electrons (Ru et al., 2021). Both elements can transform between different forms under the influence of soil conditions. There exists an antagonistic relationship between soil Cd and Se. As the soil pH increases, Cd ions are more readily adsorbed by Si–Ca–Mg oxides, thereby reducing their adsorption capacity for Se and allowing greater amounts of Se to be released.

Soil pH is one of the most critical environmental factors influencing Cd speciation, which plays a pivotal role in the *in situ* passivation remediation of heavy metals in soils (Wang et al., 2020). The blended soil conditioner contains alkaline compounds such as K, Si, and Mg, resulting in a high pH level (Fang et al., 2024). The study results indicated that the addition of soil conditioners significantly increased the soil pH, consistent with the findings of multiple studies. The higher the proportion of lime and Ca–Mg–P fertilizer, the more pronounced the increase in soil pH. This may be attributed to the high concentration of alkaline substances in the soil conditioner, which possesses strong acid-neutralizing capabilities (Wang et al., 2012).

### 4.2 Effect of soil conditioners on the Cd and Se concentrations in different organs

The roots are the most critical organs in the Cd uptake and transport of rice because they contain the highest Cd concentration. The order of average Cd concentration in vegetative organs was as follows: root > stem > leaf > seed. This result is consistent with the results of previous research (Li et al., 2018). A high Cd concentration in the roots means that only a small portion of the Cd will be transported to the other organs. Similarly, the variation in the Se concentration in different rice organs is similar to that of Cd. The concentrations of Cd and Se in different organs vary, which may be due to the nutrient demand or the transpiration intensity of these organs (Podazza et al., 2012).

The Cd concentration in the seed was significantly positively correlated with the Cd concentration in the root, stem, and leaf (*p* < 0.01). This is consistent with the results of previous studies (Lin et al., 2021; Yang et al., 2020). The antagonism between Se and Cd in soil–rice systems has been extensively demonstrated in previous pot experiments. However, the interaction between Se and Cd under soil conditioner conditions is more complicated. Our results indicated that the application of soil conditioners reduces the Cd concentration and increases the Se concentration in different organs of rice. This may be related to the soil conditioners increasing the soil pH.

### 4.3 Se-enriching and Cd-reducing effects and the associated mechanism of soil conditioners

Given the alkaline nature of the soil conditioners, they increased the soil pH and the number of negative charges and enhanced Cd sorption and Se enrichment, which further declined the plant uptake of Cd and increased the plant enrichment of Se (Chen et al., 2020). The Cd and Se concentrations showed similar patterns of variation in rice organs, which displayed a decline from the root to the seed in this study (Wang et al., 2013). This may be related to the unidirectional xylem transport of Cd and Se from the root to the aboveground parts (Xu et al., 2020). T3 and T9 decreased the Cd accumulation in the root, thus limiting its accumulation in rice seeds during maturity, indicating that lime and Ca–Mg–P fertilizer (the major components of T3 and T9) could limit the translocation of Cd to the aboveground parts in the later growth stages, probably via the decrease of root uptake. The T3 treatment group demonstrated the most effective Se enrichment and Cd reduction, the possible mechanism of which may be due to the thickening of the Casparian strips in the endodermis in the roots by the Si, Ca, and Mg ions to inhibit metal translocation, resulting in less accumulation of Cd in the aboveground parts of the plant. Furthermore, the competition of Ca^2+^ ions with Cd for exchange sites at the root surface could also decrease the Cd uptake and enrich the Se concentration (Ji-Zi et al., 2026; Liu et al., 2023; Zheng et al., 2022).

The correlation analysis between Cd and Se indicated that the presence of Se suppressed the Cd uptake by rice organs. Se promotes the synthesis of glutathione (GSH) and plant chelating peptides (PCs), which chelated with Cd, thereby reducing Cd mobility in rice plants (Zhu et al., 2024b). The transfer of Cd within rice plants was inhibited, thereby reducing the seed Cd concentration and increasing the Se concentration. This Cd-reducing and Se-enriching effect also correlated with rice growth and development (Xie et al., 2023). The T3 treatment primarily consisted of lime and Ca–Mg–P fertilizer, which not only increased the soil pH but also supplemented Ca and Mg ions into the soil, thereby enhancing the Se enrichment and Cd resistance capacity of rice.

## 5 Conclusion

In this study, the application of soil conditioners significantly increased the pH of paddy soil while simultaneously reducing the concentration of available Cd and increasing the concentration of available Se. In addition, the pH and EC are the primary factors that determine the variations in soil Cd and Se concentrations. The T3 treatment group demonstrated the most effective Se enrichment and Cd inhibition. The application of soil conditioners can effectively reduce the Cd uptake of various tissues of rice plants and promote Se accumulation. Lime and Ca–Mg–P fertilizer, as the primary components of a Cd-reducing and Se-enriching conditioner, demonstrated certain feasibility for the remediation of Se/Cd-contaminated paddy fields and for enhancing rice quality and yield.

## Data Availability

The original contributions presented in the study are included in the article/**Supplementary Material**. Further inquiries can be directed to the corresponding author.
