# Supplementary material for: Soil conditioners promote cadmium reduction and selenium enrichment in rice: formulation process, effects, and mechanisms
Source: Front Plant Sci. 2026 Aug 20;17:1887305. doi: 10.3389/fpls.2026.1887305 (PMC13538883; doi:10.3389/fpls.2026.1887305)
Supplement: Supplementary file 1 [file Table1.docx]

**Soil conditioners promote cadmium reduction and selenium enrichment in rice: Formulation process, effects and mechanisms**

Jingshang Xiao^1^, Hongqian Hou^1^, Jianhua Ji^[1]^*, Zhenzhen Lv^1^, Xianjin Lan^1^, Yiren Liu^1^, Ying Liu^2^, Long Qian^3^, Qinlei Rong^4^, Yun Liu^5^, Sijia Zuo^5^, Zhixin Luo^5^

*^1^Institute of Soil Fertilizer and Resource Environment, Jiangxi Academy of Agricultural Sciences/National Engineering and Technology Research Center for Red Soil Improvement/Key Laboratory of Acidified Soil Amelioration and Utilization, Ministry of Agriculture and Rural Affairs, Nanchang, 330200, China*

*^2^Yichun City Selenium Resource Development and Utilization Center, yinchun, 336000, China*

*^3^National Biopesticide Engineering Technology Research Center, Hubei Biopesticide Engineering Research Center, Hubei Academy of Agricultural Sciences, Wuhan, 430064, China*

*^4^College of Land Resources and Environment, Jiangxi Agricultural University, Nanchang, 330200, China*

*^5^Jiangxi Purifeng Ecological Technology Co., Ltd.* *Changnan Industrial Park, Nanchang, 330001‌, China*

**Experimental Materials and Design**

The tested rice varieties were the main varieties suitable for planting locally, the late rice variety was Yongyou 1538, and the basic seedlings were 18,000 roots. Lime was provided by Shanggao Xuelin Lime Powder Factory, with calcium carbonate content≥95%; Zeolite was provided by Zhejiang Shenshi Mining Co., Ltd., and particle size was 40-100 mesh; Ca-Mg-P fertilizer was provided by Hubei Jinmingzhu Chemical Co., Ltd., P_2_O_5_≥12%,CaO≥20% MgO ≥4%,SiO_2_≥20%; Sodium humate was provided by Anhua Biotechnology Co., Ltd., Sodium humate content ≥50%, pH=8.7; Compound fertilizer (N-P_2_O_5_-K_2_O 15-15-15) was provided by China Salt Anhui Hongsifang Co., Ltd.,; Urea (N 46%) was provided by Haoyuan Chemical Co., Ltd.,; Potassium chloride (K_2_O 60%) was provided by Sinochem.

The experimental design was based on the uniform experimental design (UED) methodology proposed by Kaitai Fang, utilizing regression-derived models for predictive analysis and parameter optimization. Field test with lime, Ca-Mg-P fertilizer, zeolite, sodium humate as uniform design factors, each factor was 9 levels, 4 factors 9 level uniform design table U9 (94) design 9 tests, the treatment without any conditioner was also set as control (CK), 10 treatment (see table 1), each treatment three repeated, a total of 30 plots, The area of each plot was 30 m^2^ (5 m×6 m), random group arrangement. The plots were separated by ridges 0.45 m deep and 0.5 m wide, which were wrapped in plastic film and drained independently. Field management of the plots was consistent with the farmers' customary fertilization treatments.

The same amount of fertilizer and application method was applied to the late rice trials. The application rates of N, P and K were 180, 90, 135 kg·hm^-2^ as urea and compound fertilizer in tillering stage; and 75 kg·hm^-2^ of potassium chloride and 75 kg·hm^-2^ of urea were applied at the stage of differentiation of young spikes. All soil conditioner materials were used as base fertilizer, and all fertilizers were applied on the surface.

Potted trials were conducted for late rice cultivation using the same varieties as in the field trials. Four experimental treatments were established: limestone powder, calcium-magnesium phosphate fertilizer, zeolite, and sodium humate. The control (CK) received no conditioning agent. A total of five treatments were tested; each replicated three times across 15 pots. Each pot contained 10 kg of soil and three rice plants. Limes was applied at 3750 kg·hm⁻², while Ca-Mg-P fertilizer, zeolite, and sodium humate were each applied at 7500 kg·hm⁻². Nitrogen fertilizer application rate was 1. 5 times that of the field trial. Phosphorus and potassium fertilizer rates, application methods, and timing were identical to the field trial. All soil conditioner materials were incorporated as basal fertilizer.

OC: 1 g samples were placed into a 50 ml colorimetric tube, added 0.5g HgSO_4_, 5 ml K_2_Cr_2_0_7_ solution, 7.5 ml concentrated sulfuric acid. The colorimetric tubes were shaken well and then placed in a drying cabinet (DHG-9053A, Yiheng, China) and heated at 150°C for 4 h. Remove the colorimetric tube and add deionized water to the scale, shake well and leave overnight and determine the reaction solution at a wavelength of 485 nm against a reagent blank solution.

TN : Weigh 1.0 g of pre-sieved air-dried soil directly into a dedicated digestion tube, add one pre-mixed Kjeldahl catalyst tablet and 5 mL concentrated sulfuric acid, heat the mixture on a 380℃ digestion furnace for 75 minutes until the solution turns fully clear, let it cool for 10 minutes, connect the tube to the pre-warmed integrated Kjeldahl distillation unit, add excess 40% NaOH automatically, distill the released ammonia directly into pre-prepared boric acid indicator solution for 5 minutes, then titrate the absorbed solution with standardized 0.01 mol/L HCl to the stable color end point, run one blank per batch of samples, and calculate the soil total nitrogen content directly without extra constant-volume steps.

AP: AP was extracted from samples using a 0.5 mol L^-1^ NaHCO_3_ solution, added 1 teaspoon of activated carbon (phosphorus-free), shake the reaction for 30 min. After filtration using phosphorus-free filter paper, 10 ml of supernatant was taken for analysis. The AP content of the extract was then determined by molybdenum antimony colorimetric method.

AK: Weigh 5g of 2mm-sieved air-dried soil, add 50mL of 1mol/L pH7.0 ammonium acetate solution directly, shake for 30 minutes and filter to get the clear extract, preheat the flame photometer for 15 minutes, run the pre-configured 0-40μg/mL potassium standard series to finish the calibration, then directly test the filtrate to read the concentration, and calculate the final soil available potassium content in one step without extra solution transfer or secondary dilution.

**Cd and Se measurement procedure**

**Procedure for the extraction of total Se and total Cd from soil**

Weigh 0.2 g of the sample on an analytical balance, add a small amount of water to moisten the sample, and add 10 mL of HNO_3_-HClO_4_ (4:1); tighten the jar lid, place the jar in the digestion apparatus; pre-digest at 120°C for 30 minutes, then proceed to microwave digestion using the following program: heat up to 130°C for 5 minutes, followed by a 3-minute hold; 150°C, heating time 3 min, holding time 10 min; 180°C, heating time 3 min, holding time 30 min. Allow to cool to 60°C, then dilute to a final volume of 25 mL with deionized water, shake well, and prepare for analysis.

**Procedure for extracting available Se and Cd from soil**

**Procedure for extracting available Cd**

Weigh 10.0 g of air-dried soil sample that has been passed through a 2 mm sieve and place it in a 250 mLconical flask. Add 20 mL of DTPA extractant, and shake at a constant temperature of 25°C for 2 hours, maintaining a shaking speed of 180 r/min. Transfer the mixture to a centrifuge and centrifuge at 3000 r/min for 10 min. Then, collect the supernatant and filter it through a 0.45 μm water-based filter membrane to obtain the sample solution.

**Procedure for extracting available Se**

Weigh 5.0 g of air-dried soil sample that has been passed through 2 mm sieve and place it in a 100 mL stoppered conical flask. Add 25 mL of 0.5 mol/L MgCl_2_ and shake at a constant temperature of 25°C for 30 minutes. Transfer an appropriate amount of the extract to a 50 mL volumetric flask, add concentrated hydrochloric acid, and heat to reduce hexavalent Se to tetravalent Se. After cooling, make up to volume for analysis.

**ICP-OES operation steps**

Power on the ICP-OES system with argon pressure adjusted to 0.6 MPa, start the cooling water and complete the 3-minute quick self-check, ignite the plasma under optimized working conditions of 1300 W RF power, 15 L/min plasma gas flow and 0.7 L/min nebulizer flow, run the pre-configured 3-point matrix-matched standard series to finish calibration, which ensures the instrument reaches a typical detection limit of 0.1-5 μg/L for most target elements, test all pre-digested samples continuously, rinse the sample line with 2% dilute nitric acid for 2 minutes after detection, then extinguish the plasma and shut down all supporting equipment in sequence.

**ICP-OES detection limit**

Summary Table of Detection Limits and Quantitation Limits for Determination of Elements by Analytical Method Unit: μg/kg

| **Element** | **Detection Limit** | **Quantitation Limit** | **Element** | **Detection Limit** | **Quantitation Limit** |
| --- | --- | --- | --- | --- | --- |
| Silver (Ag) | 0.04 | 0.16 | Hafnium (Hf) | 0.03 | 0.12 |
| Aluminum (Al) | 1.15 | 4.6 | Holmium (Ho) | 0.03 | 0.12 |
| Arsenic (As) | 0.12 | 0.48 | Indium (In) | 0.03 | 0.12 |
| Gold (Au) | 0.02 | 0.08 | Iridium (Ir) | 0.04 | 0.16 |
| Boron (B) | 1.25 | 5 | Potassium (K) | 4.5 | 18 |
| Barium (Ba) | 0.2 | 0.8 | Lanthanum (La) | 0.02 | 0.08 |
| Beryllium (Be) | 0.04 | 0.16 | Lithium (Li) | 0.33 | 1.32 |
| Bismuth (Bi) | 0.03 | 0.12 | Lutetium (Lu) | 0.04 | 0.16 |
| Calcium (Ca) | 6.61 | 26.4 | Magnesium (Mg) | 1.94 | 7.76 |
| Cadmium (Cd) | 0.05 | 0.2 | Manganese (Mn) | 0.12 | 0.48 |
| Cerium (Ce) | 0.03 | 0.12 | Molybdenum (Mo) | 0.06 | 0.24 |
| Cobalt (Co) | 0.03 | 0.12 | Sodium (Na) | 6.36 | 25.4 |
| Chromium (Cr) | 0.11 | 0.44 | Niobium (Nb) | 0.02 | 0.08 |
| Cesium (Cs) | 0.03 | 0.12 | Neodymium (Nd) | 0.04 | 0.16 |
| Copper (Cu) | 0.08 | 0.32 | Nickel (Ni) | 0.06 | 0.24 |
| Dysprosium (Dy) | 0.03 | 0.12 | Phosphorus (P) | 19.6 | 78.4 |
| Erbium (Er) | 0.02 | 0.08 | Lead (Pb) | 0.09 | 0.36 |
| Europium (Eu) | 0.04 | 0.16 | Palladium (Pd) | 0.02 | 0.08 |
| Iron (Fe) | 0.82 | 3.28 | Praseodymium (Pr) | 0.04 | 0.16 |
| Gallium (Ga) | 0.02 | 0.08 | Platinum (Pt) | 0.03 | 0.12 |
| Gadolinium (Gd) | 0.03 | 0.12 | Rubidium (Rb) | 0.04 | 0.16 |
| Germanium (Ge) | 0.02 | 0.08 | Rhenium (Re) | 0.04 | 0.16 |
| Rhodium (Rh) | 0.03 | 0.12 | Thorium (Th) | 0.05 | 0.2 |
| Ruthenium (Ru) | 0.05 | 0.2 | Titanium (Ti) | 0.46 | 1.84 |
| Antimony (Sb) | 0.15 | 0.6 | Thallium (Tl) | 0.02 | 0.08 |
| Scandium (Sc) | 0.2 | 0.8 | Thulium (Tm) | 0.04 | 0.16 |
| Selenium (Se) | 0.41 | 1.64 | Uranium (U) | 0.04 | 0.16 |
| Samarium (Sm) | 0.04 | 0.16 | Vanadium (V) | 0.08 | 0.32 |
| Tin (Sn) | 0.08 | 0.32 | Tungsten (W) | 0.43 | 1.72 |
| Strontium (Sr) | 0.29 | 1.16 | Yttrium (Y) | 0.04 | 0.16 |
| Terbium (Tb) | 0.05 | 0.2 | Ytterbium (Yb) | 0.05 | 0.2 |
| Tellurium (Te) | 0.05 | 0.2 | Zinc (Zn) | 0.67 | 2.68 |

**Table S1** Basic physical and chemical properties of the test soil

| Region | Jie guan |
| --- | --- |
| Organic matter (g/kg) | 35.4 |
| Total Nitrogen (g/kg) | 2.01 |
| Alkaline nitrogen (mg/kg) | 193.38 |
| Available Phosphorus (mg/kg) | 21.76 |
| Available Potassium (mg/kg) | 173.06 |
| pH | 5.48 |
| Total Cadmium (mg/kg) | 0.39 |
| Total Selenium (mg/kg) | 0.46 |
| Available Cadmium (mg/kg) | 0.18 |
| Available Selenium (mg/kg) | 0.051 |

**Table S2** Regression prediction features

| Sample | Observed values | | Fitted values | | Fitting error |
| --- | --- | --- | --- | --- | --- |
| 1 | 36.9481 | 39.1887 | | -1.0848 | |
| 2 | 49.813 | 48.7566 | | 2.3845 | |
| 3 | 54.9069 | 56.8049 | | -1.6095 | |
| 4 | 36.3086 | 34.4712 | | 0.1864 | |
| 5 | 42.9106 | 42.4218 | | 0.6097 | |
| 6 | 47.011 | 47.9096 | | 0.3514 | |
| 7 | 34.845 | 37.2749 | | -0.6562 | |
| 8 | 41.5558 | 42.181 | | -0.5899 | |
| 10 | 46.0147 | 42.9664 | | 0.4602 | |

1. * Corresponding author: Jianhua Ji; Institute of Soil Fertilizer and Resource Environment, Jiangxi Academy of Agricultural Sciences, Nanchang 330200, China. Email: [*420200426@qq.com*](mailto:420200426@qq.com); Mobile telephone is +86-13576990955.
